# The Mediterranean mussel *Mytilus galloprovincialis*: responses to climate change scenarios as a function of the original habitat

**DOI:** 10.1093/conphys/coaa114

**Published:** 2021-02-04

**Authors:** Jihene Lassoued, X A Padín, Luc A Comeau, Nejla Bejaoui, Fiz F Pérez, Jose M F Babarro

**Affiliations:** 1 Instituto de Investigaciones Marinas, IIM-CSIC, Eduardo Cabello 6, 36208 Vigo (Pontevedra), Spain; 2 Institut National Agronomique de Tunisie, INAT, Université de Carthage, 43 Avenue Charles Nicolle, 1082 Tunis, Tunisia; 3 Laboratory of Blue Biotechnology and Aquatic Bioproducts (B3Aqua), Institut National des Sciences et Technologies de la Mer (INSTM), Annexe La Goulette Port de Pêche, 2060 La Goulette, Tunisia; 4 Fisheries and Oceans Canada, Gulf Fisheries Centre, 343 Université Avenue, Moncton, New Brunswick E1C 9B6, Canada

**Keywords:** Marine mussel, ecophysiology, climate change

## Abstract

The impact of simulated seawater acidification and warming conditions on specimens of the mussel *Mytilus galloprovincialis* locally adapted to very distinct, widely separated sites in the Mediterranean Sea (Tunisia) and Atlantic Sea (Galicia, NW Spain) was evaluated in relation to key behavioural and eco-physiological parameters. Over the 2-month exposure to the experimental conditions, mussels were fed optimally to ensure that there are no synergistic interactions between climate change drivers and energetic status of the individuals. In general, regardless of origin (Atlantic or Mediterranean), the mussels were rather resilient to acidification for most of the parameters considered and they were able to grow in strongly acidified seawater through an increased feeding activity. However, shell strength decreased (40%) consistently in both mussel populations held in moderately and highly acidified seawater. The observed reduction in shell strength was not explained by slight alterations in organic matter, shell thickness or aragonite:calcite ratio. The combined effects of high acidification and warming on the key response of byssus strength caused a strong decline in mussel performance, although only in Galician mussels, in which the valve opening time decreased sharply as well as condition index (soft tissue state) and shell growth. By contrast, the observed negative effect of highly acidified scenario on the strength of Tunisian mussel shells was (partly but not totally) counterbalanced by the higher seawater temperature. Eco-physiological and behavioural interactions in mussels in relation to climate change are complex, and future scenarios for the ecology of the species and also the feasibility of cultivating them in Atlantic and Mediterranean zones are discussed.

## Teaser

The responses of two very distinct populations of the same marine mussel species *Mytilus galloprovincialis*, and inhabiting the Atlantic and Mediterranean Seas, were evaluated facing climate change scenarios with regard to *p*CO_2_ and temperature drivers. The potential threats and resilient behaviour of individuals according to their original habitat were drawn and discussed.

## Introduction

Approximately one-third of the total anthropogenic CO_2_ emissions have been absorbed by the ocean (Sabine *et al.*, 2004), in turn triggering a process called ocean acidification (OA) (Orr *et al.*, 2005; Gattuso *et al.*, 2015). Anthropogenic activities have almost doubled the pre-industrial concentrations of atmospheric CO_2_ (280 μatm *p*CO_2_) to around the actual 408 μatm *p*CO_2_ (https://www.noaa.gov/). The rise in *p*CO_2_ that has caused OA is measured as a decline in seawater pH, accompanied by a decrease in both the carbonate ion (CO_3_^2−^) concentration and the saturation states (Ω) of various calcium carbonates forms (Zeebe and Westbroek, 2003). Contemporary surface ocean pH has decreased by 0.1 units since pre-industrial time (Raven *et al.*, 2005). The *p*CO_2_ is expected to rise above 1100 μatm by 2100, which would lead to a decrease in seawater pH of approximately 0.3–0.4 units (IPCC, 2014). In addition, the IPCC (2014) has also highlighted the fact that the upper 75 m of oceans has warmed at an average rate of ~0.1°C per decade over the past 40 years. Modelling studies have predicted that the mean global ocean temperature may increase within 1–4°C by 2100 (Doney *et al.*, 2012), although trends may differ regionally, seasonally and inter-annually.

Calcifying organisms are at a greater risk than other species as a consequence of the large amount of energy allocated to shell deposition and changes in acid–base equilibrium, energy budgets and CaCO_3_ saturation state by OA (Orr *et al.*, 2005; Fabry *et al.*, 2008; Nienhuis *et al.*, 2010; Gazeau *et al.*, 2013). Most studies have highlighted the negative effects of OA on calcification rates, biomineralization or shell functionality (Gazeau *et al.*, 2007; Fabry *et al.*, 2008; Hendriks *et al.*, 2010; Kroeker *et al.*, 2010; Gaylord *et al.*, 2011; Duarte *et al.*, 2015). This effect is derived from the fact that shell (growth) maintenance may compromise shell functioning through energetically costly metabolic compensation for the increased shell dissolution that occurs under OA (Mackenzie *et al.*, 2014). Nevertheless, neutral or even positive effects of OA have also been described regarding specific metabolic responses (Ries *et al.*, 2009; Melzner *et al.*, 2011; Fernández-Reiriz *et al.*, 2012; Range *et al.*, 2012; Gazeau *et al.*, 2014; Mackenzie *et al.*, 2014), especially when food resources are optimal during the maintenance of individuals through increasing feeding activity (Lassoued *et al.*, 2019). These very variable responses may be explained by the distinct pH-regulation values considering species-specific capacities (Fabry *et al.*, 2008) and also different life-history stages of the same species, e.g. higher sensitivity in early life stages (Kroeker *et al.*, 2013). In general, biological responses to high *p*CO_2_ conditions are variable and complex, species-specific and strongly dependent on the environmental variability experienced by species in their local habitats (Osores *et al.*, 2017).

Temperature is an important driver of physiological adaptation in marine species in response to stress through its impact on metabolism and growth (Gazeau *et al.*, 2013). As marine organisms exist in conditions close to their thermal compensatory capacity (Somero, 2002), seawater warming (SW) will impact all physiological activities and therefore survival and other ecological interactions. Both OA and SW may vary simultaneously and interactively in response to a particular climate change scenario (Li *et al.*, 2015), thus implying that a combination of biological impacts may also occur (Kroeker *et al.*, 2013). Indeed, environmental changes occur simultaneously and tests are required for multiple stressors. The interactions between climate change drivers vary and include additive effects (Hiebenthal *et al.*, 2013), synergistic effects (Ivanina *et al.*, 2013) and antagonistic effects (one stressor may offset the effect of the other) (see Folt *et al.*, 1999). For instance, OA reduces the thermal tolerance of calcifiers, e.g. marine crabs (Walther *et al.*, 2009), whereas SW reduces the negative effects of OA in sea urchins (Sheppard Brennand *et al.*, 2010) but may enhance the sensitivity of calcifiers to OA regarding growth, biomineralization and survival (Kroeker *et al.*, 2013). The combination of high temperature and elevated CO_2_ was found to significantly decrease shell hardness in *Crassostrea virginica* and *Mercenaria mercenaria*, which suggests changes in biomineralization processes(Ivanina *et al.*, 2013). Previous studies have also shown that marine mussels (*Mytilus* spp.) are more sensitive to SW than to OA scenarios (Mackenzie *et al.*, 2014, with *M. edulis*, and Gazeau *et al.*, 2014, with *M. galloprovincialis*). Nonetheless, in-depth studies highlighting the effects of OA and SW for the same species inhabiting distinct environments are required in order to clarify the impact of local environmental heterogeneity on marine calcifiers.

In marine calcifiers such as mussels, the protective calcium carbonate shell and the byssus apparatus appear to be key target variables for assessing the consequences at the individual level and also the hypothetical interactions within ecosystems. Any strategy that can cope with decreased seawater pH or any other stress factor will be energetically demanding. The energetic costs of both biomineralization and byssogenesis processes are consistent with relatively higher maintenance ranges, and extra stressors such as food scarcity may further exacerbate the negative impact of OA (Melzner *et al.*, 2011; Mackenzie *et al.*, 2014; Lassoued *et al.*, 2019).

The Mediterranean mussel *M. galloprovincialis* inhabits a wide range of geographical zones as it is distributed throughout the whole Mediterranean Sea and also the Atlantic coast of Spain. A greater decrease in pH has been reported for the Mediterranean Sea (−0.0028 pH_T_/year) than for other areas such as the South Pacific and North Atlantic (−0.0013 and − 0.0026, respectively) for 2007–15 (Bates *et al.*, 2014; Kapsenberg *et al.*, 2017). This decrease would, in turn, make the former area more vulnerable to further climate change. *Mytilus galloprovincialis* is currently one of the most commonly cultivated bivalve species in the world. Global production of the mussels has increased drastically in the past 50 years, reaching around 1 million tons in 2011. China and Spain (98% on the Atlantic coast) are the top producers, producing ∼700 000 and ∼250 000 tons/year, respectively (FAO, 2016). This bivalve is also one of the most common cultivated marine species in the Mediterranean Sea (FAO, 2014). In the north of Tunisia, the main mussel breeding site is the lagoon of Bizerte (37° 11′ 48″ N, 9° 51′ 23″ E), where the local industry produces on average 115 tons/year (DPGA, 2017). Seawater temperature varies widely from 9.8 to 28°C, although it can reach up to 30.8°C in summer (Béjaoui *et al.*, 2016). The maximum summer temperature in the northwestern Mediterranean Sea increased by ~1°C between 2002 and 2010, relative to the 1980–2000 average (Marbá and Duarte, 2010), and rapid warming of 2.8 ± 1.1°C is expected by the end of the century (Jordá *et al.*, 2012). SW can kill organisms such as *M. galloprovincialis* (Anestis *et al.*, 2007; Gazeau *et al.*, 2014) during the summer and autumn periods when the seawater temperature rises above 26–27°C. Shellfish farmers are being forced to sell their products in early summer to prevent harvesting losses or mass mortalities (Ramón *et al.*, 2005).

Spanish production is predominantly on the Atlantic coast: mussels are currently most successfully cultivated in the Ría de Arousa (NW Spain), the largest natural embayment of the Rías Baixas (42° 30′ 00″ N, 8° 56′ 00″ W), on the western Atlantic coast (average 250 000 tons/year; FAO, 2016). The seawater temperature varies from 12°C to 19°C throughout the year (Álvarez *et al.*, 2005), although specific events during recent summers have occasionally increased the temperature of surface waters to 20°C.

Beyond huge differences in biomass production, the latter two geographical sites where the mussel *M. galloprovincialis* is distributed and cultivated provide a good opportunity to study the importance of mussel origin and endogenous adaptation to distinct habitats in relation to a number of eco-physiological responses. These responses may represent key strategies for survival and fitness facing stressful conditions such as OA and SW. In this laboratory-based study, we assessed the effects of *p*CO_2_-driven acidification and the simultaneous highly acidified and SW scenarios on byssus and shell functionality and on the behavioural and physiological performances of *M. galloprovincialis*, to ensure prediction of the impact on ecosystem structure and functioning. The different thermal windows of the marine mussels under study (owing to their different origins) enable inference of their potential tolerance or resilience in response to the predicted climate change scenarios.

## Materials and methods

### Collection and maintenance of mussels

Juvenile specimens of the mussel *M. galloprovincialis* (30.69 ± 0.28-mm shell length, mean ± SE) were sampled from suspended cultivation ropes in two subtidal locations: the Ría de Arousa (NW Spain) and Bizerte Lagoon (North Tunisia). The specimens were transported to the IIM-CSIC laboratory (Vigo, Spain) where epibionts were removed from the shells and byssal threads were carefully cut from the ventral margin to prevent damaging the byssus gland or foot organ. The mussels were maintained in an open flow-through seawater system held at 15°C with a 12:12-h (light:dark) photoperiod cycle. The system supplied ~1 mg/l of seston as a mixture of two phytoplankton cultures of *Isochrysis galbana* clone T-Iso and *Rhodomonas lens* (50–50% in weight).

### Experimental design

The mussels were acclimated for 10 days in the laboratory before being exposed to a modified carbonate system for 2 months. Four experimental scenarios were considered: seawater temperature = 15°C and *p*CO_2_ concentration = 1200 μatm (i.e. low temperature, strong acidification); seawater temperature = 24°C and *p*CO_2_ concentration = 1200 μatm (i.e. high temperature, strong acidification); seawater temperature = 15°C and *p*CO_2_ concentration = 800 μatm (i.e. low temperature, intermediate acidification); and seawater temperature = 15°C and *p*CO_2_ concentration = 400 μatm (low temperature, low acidification; control scenario). Four 9-l tanks (34 × 23 × 19 cm; length × width × height) were used for each of the four scenarios: three tanks with mussels plus one control tank without mussels. Two distinct pieces of glass (24 × 10 cm; length × width each) were placed independently on the bottom of each tank that will serve as a substrate for the two mussel populations under study. Another piece of glass (24 × 20 cm; length × width each) was placed below those two pieces of glass (substrate for mussels) with a vertical piece of glass (24 × 4 cm; length × height) that served to maintain separated and independent both mussel populations though chemical characteristics of seawater and food particles concentration supplemented were the same in the whole tank (previously tested with PAMAS equipment by monitoring left–right and bottom–up transects in the tank; see CR measurement below). For each experimental scenario, 18 juvenile specimens from each origin were placed in one half of the tank. The mussels in both sides of the tanks were able to form clusters freely and attach to glass plates (24 × 10 cm; length × width) placed on the bottom of the tanks without physical interactions between the two sides of the tank, while providing easy access to the whole attached population on the glass plates. Air bubbling and optimal food regime were added to ensure homogeneous distribution of phytoplankton cells with sufficient concentration (see Section: Collection and maintenance of mussels) to avoid food availability restrictions or low feeding ratios that could make mussels to compete for scarcity of food resources. Seawater enriched with microalgae (*I. galbana* clone T-Iso and *R. lens*) and held in three 200-l header tanks was supplied to the experimental tanks containing mussels via peristaltic pumps (ISMATEC^R^). The header tanks with food, the peristaltic pumps and all tanks with and without mussels were connected by randomly arranged tubing. The mussels were allowed to establish primary attachment to experimental units for 1 week, e.g. glasses on the bottom, and any individual that did not produce byssus filaments were removed and replaced with other mussels.

Atmospheric air and pure CO_2_ gas were previously mixed in separate tanks before being constantly bubbled through the experimental tanks. Gas concentrations were continuously logged via LI-COR 6262 CO_2_ gas analyzers, and the measurements were used to adjust the gas mixture through software-controlled solenoid valves. Seawater salinity was recorded weekly (8410 Portasal; Guildline Instruments). Seawater samples were also collected every week for analysis of total alkalinity (A_T_) (in duplicate). A_T_ was measured at one end point, with an automatic potentiometric titrator (809 Titrando and 800 Dosino; Metrohm) and a combined glass electrode (Pérez and Fraga, 1987a). The samples were transferred with the aid of a Knudsen pipette (~50 ml) to an open Erlenmeyer flask for potentiometric titration with 0.1 M HCl. The final titration volume was determined by two pH readings after the end point of 4.45 was reached (Mintrop *et al.*, 2000). Certified reference material (CRM; batch #163) for CO_2_ in seawater (provided by A. Dickson, Scripps Institution of Oceanography, University of California; San Diego, CA, USA) was used to quantify the analytical error. Seawater samples were collected twice weekly from all tanks and placed directly in optical glass spectrophotometric cells (volume: 28 ml; path length: 100 mm) for pH determination. The cells were held in a thermostatic bath at 25°C for ~1 h before analysis. The pH was measured by the spectrophotometric method described by Clayton and Byrne (1993). More detailed information about the analytical methods is provided in a previous article (Lassoued *et al.*, 2019). Calculations were carried out using the equations proposed by Dickson *et al.* (2007), who included a correction factor for the difference between seawater and the acidity indicator. Seawater A_T_, salinity, temperature and pH data were used to calculate other seawater parameters by using CO2SYS software (van Heuven *et al.*, 2011) and applying dissociation constants for carbonic acid (Lueker *et al.*, 2000) and the constants for borate and hydrofluoric acid (Pérez and Fraga, 1987b; Dickson, 1990).

Experimental diets were produced by manipulating the two microalgae cultures used for maintenance (*I. galbana* clone T-Iso and *R. lens*) to yield a particle load of ~1.2 mg/l. Food was supplied to the mussels at a continuous flow rate of 9 ml min^−1^ from the header tanks. This flow rate allowed removal of ammonium and other waste products due to mussel metabolism or bacteria. Seawater chemistry parameters controlled throughout the experimental period are shown in Table 1.

**Table 1:** Experimental seawater chemistry parameters: salinity, pH, pCO_2_ (μatm), bicarbonate (HCO_3_^−^) and carbonate (CO_3_^2−^), total alkalinity (TA), calcite and aragonite saturation states (ΩCa and ΩAr)

	Target *p*CO_2_/°C	T (°C)	Salinity (ppt)	pH	pCO_2_ (μatm)	HCO_3_^−^ (μmol/kg)	CO_3_^2−^ (μmol/kg)	TA (μmol/Kg)	ΩCa	ΩAr
Treatment	400/15	15.4 ±0.4	34.53 ±1.3	7.992 ±0.031	439 ±32	1846 ±64	136 ±14	2186 ±86	3.25 ±0.31	2.09 ±0.20
	800/15	15.3 ±0.4	34.50 ±1.3	7.747 ±0.028	836 ±32	1997 ±53	83 ±13	2205 ±73	1.99 ±0.29	1.28 ±0.19
	1200/15	14.9 ±0.4	34.52 ±1.3	7.625 ±0.030	1134 ±48	2052 ±60	64 ±9	2212 ±78	1.52 ±0.20	0.97 ±0.13
	1200/24	24.8 ±0.4	34.70 ±1.2	7.644 ±0.020	1118 ±35	1980 ±53	94 ±7	2211 ±68	2.25 ±0.16	1.48 ±0.10
Control	400/15	15.5 ±0.4	34.72 ±1.3	8.002 ±0.025	448 ±51	1923 ±66	146 ±6	2286 ±80	3.48 ±0.14	2.24 ±0.09
	800/15	15.6 ±0.6	34.60 ±1.3	7.767 ±0.025	822 ±56	2055 ±59	90 ±6	2280 ±71	2.16 ±0.13	1.39 ±0.09
	1200/15	15.5 ±0.7	34.55 ±1.3	7.643 ±0.036	1116 ±104	2101 ±72	69 ±10	2274 ±88	1.65 ±0.24	1.06 ±0.16
	1200/24	23.8 ±0.6	35.24 ±1.3	7.683 ±0.029	1056 ±86	2065 ±58	103 ±8	2318 ±68	2.46 ±0.18	1.62 ±0.12

Values are means ±standard error.

### Mussel responses

#### Clearance rate

The feeding activity of the mussels was measured as the clearance rate (CR) and the volume of water cleared of known suspended algal cells volume (T-ISO and *R. lens*) was determined according to Cranford *et al.* (2016). These parameters were measured using a calibrated PAMAS laser particle counter (Model S4031GO Seawater, 2017: 421-0289). As the phytoplankton cells used in this study are within the known size range distribution, the PAMAS was initially calibrated to determine the particle size distribution between 1 and 20 μm. Nevertheless, the actual range used to determine CRs was established at 4.5–7.5 μm, according to the highest retention efficiency values for the mussels (above 4 μm; Møhlenberg and Riisgård, 1978). The CR was determined once a week during the experimental period, for each mussel population. The mussels were left undisturbed on the bottom of the tanks, i.e. attached to glass substrates. As glass substrates with mussels from both origins are independent from each other but in the same tank (see above), CR measurements for each population were also carried out independently by removal of one of the populations (simply taking the glass with the clusters formed out of tank) during monitoring of the other. Responses of the same population in the different tanks were monitored first for the single experimental treatment in order to allow the removed population from the same tank to acclimate back in the original tank at least for 2 h before monitoring CR. A known volume of phytoplankton cells of the two microalgae used in the experiment was added to each tank, and PAMAS measurements began after 5 min, thus ensuring good mixing in the tank. The homogeneity of phytoplankton concentration within experimental tanks was aided by the CO_2_-air agitation system and confirmed by noting the concentration of microalgae across different sections of the tank (right to left and bottom to top). The PAMAS was immediately set up to monitor the decrease in the number of algal cells over time during 20 min. Control tanks with no mussels were also monitored to identify any reduction in phytoplankton due to deposition on the bottom and for correcting the tanks containing mussels. Any decrease was subsequently corrected by the slope of the control tank. The CR was determined from the linear decrease in algal concentration (verified as a straight line in a semi-log plot) over time, by applying the formula proposed by Riisgård and Seerup (2003): *CR* =   *V*/*n*, where *V* is the volume of water in the tank,  is the slope of the regression line in a semi-ln plot of the reduction in algal concentration with time in the aquarium with mussels and *n* is number of mussels per tank. Only regression coefficients (r^2^) greater than 0.9 were considered in the data analysis.

#### Valve opening behaviour

For each of the four experimental scenarios (*p*CO_2_ and temperature treatments), four specimens in two of the three tanks containing mussels were connected to a non-invasive high-frequency valvometry system (*n* = 8 mussels per experimental condition). The system was constructed by glueing (with cyanoacrylate glue) a coated Hall element sensor to a valve and then glueing a magnet (4.8-mm diameter × 0.8-mm height) to the other valve, on exactly the opposite side of the Hall sensor. For other specifications of this technique, see Comeau *et al.* (2018) and Lassoued *et al.* (2019) for similar experimental set-up. Valve opening was monitored at an interval of one measurement per second throughout the whole experimental period. Due to logistic constraints, valve opening was monitored in three experimental scenarios, e.g. the control scenario (15°C, 400-μatm *p*CO_2_,), the low temperature, strong acidification scenario (15°C, 1200-μatm *p*CO_2_) and the high temperature, strong acidification scenario (24°C, 1200-μatm *p*CO_2_).

A relative valve-opening metric for each mussel was computed as the percent of the maximum recorded opening amplitude. The results were partitioned into 10 equal ranges (0–10%, 10–20%, 20–30%...) of the opening amplitude. Percent occurrence was calculated as the number of observations within each opening range divided by the total number of observations (Tran *et al.*, 2010). This preliminary analysis revealed that percent occurrences were normally distributed between 10 and 100% of opening amplitude; however, a spike in occurrences below 10% of the amplitude was indicative of a distinct behaviour, which we categorized as shell closure. Hence, for any given individual, all amplitudes falling below 10% of the maximum recorded amplitude were considered as shell closures. This decision was further substantiated with accuracy and precision testing of the Hall technique using plastic wedges of known dimensions mimicking shell opening amplitudes. In keeping with this information, we finally calculated a simple behavioural metric for each individual: the percentage of time shells were opened (≥10% amplitude) over the course of the 2-month experiment.

#### Byssus strength

Mussel detachment force measurements were conducted with mussels immersed in water to avoid modification of the mechanical properties of the byssus by air-dryness conditions. Care was taken to avoid disturbing neighbouring mussels when dislodging one individual. Individuals that were immediately adjacent to those selected for dislodgement were not considered for trials if they had interconnected byssus threads. Byssal detachment strength was measured by clamping the mussel shell to a spring scale [Digital Force Gauge DN431 (Caceres, Spain) with peak hold measurement, resolution of 0.01 N] using custom-made forceps. Clusters of individual mussels required a certain flexibility of the digital force equipment so as to be able to connect to the desired mussel within aggregations. Therefore, a byssus detachment force of the whole individual living in aggregations is referred to rather than the adhesion force or byssus failure mode (Burkett*et al.*, 2009). The spring scale was pulled perpendicular to the substratum until dislodgement occurred (Bell and Gosline, 1997; Babarro and Carrington, 2011), which ensures that most of byssus threads secreted are under tension.

#### Shell characteristics: shell thickness index, strength, organic matter and aragonite:calcite ratio

The same individuals used to measure the byssus strength within the mussel cluster were also used to determine the shell thickness index (STI) and the destructive value, e.g. compressive strength. The STI was calculated according to the formula STI = 1000 × dry shell wt/[L(H^2^ + W^2^)^0.5^ × π/2], where L, H and W are the length, height and width, respectively, of the shell (Freeman *et al.*, 2009) measured with a digital vernier calliper (±0.01 mm). For estimation of shell weight, the mussels were sacrificed, the tissue was removed and the shells were blotted dry with paper towels and weighed on a sartorius digital precision balance (±0 0.01 mg).

The left valve was used for all compressive strength analyses. The force required to crack the horizontally arranged shells was measured in a universal testing machine (Instron 5566), with 1-kN load cell and at a rate of 2 mm s^−1^ (see Babarro and Abad, 2013 for other specifications). Shell strength was calculated from the maximum force measured in the curves and was then normalized by the shell thickness measured with a micro-calliper (Mitutoyo 0–25 ± 0.01 mm) at the highest point of the shell (when placed on a horizontal plane) where the force was applied.

Three mussels were collected from the other clusters in each tank after the byssus strength was determined. The organic matter (OM) and calcite and aragonite contents of the shells were determined according to Addadi *et al.* (2006). The OM content was determined by the gravimetric method, by calculating the loss of organic weight after calcination at 500°C for 48 h. Calcium, magnesium and strontium were extracted with hydrochloric acid and the contents were measured by inductively coupled plasma optical emission spectrometry. Semi-quantitative phase proportions were determined for mixtures of carbonate minerals by powder x-ray diffraction, as described by Davies and Hoofer (1963) for calcite and aragonite. All elemental and compound analyses were performed by the CACTI analytical services (University of Vigo).

#### Condition index and specific growth rate

Three mussels from each of the three tanks per experimental treatment were used to determine the condition index (CI) values. The CI was calculated as DW_soft tissue_/DW_shell_) × 100, where DW_soft tissue_ is the dry weight of the soft tissue and DW_shell_ is the dry shell weight (Freeman, 1974).

The length, width and height of all individuals were measured at the beginning and the end of the experiment. The mean (±0.01 mm) values for each mussel cluster in each tank were then obtained. The specific growth rate (SGR) of the mussel cluster was calculated from the mean values, as follows: SGR = ln (F_length_/I_length_) × *t*  ^−1^, where F_length_ and I_length_ represent the mean shell lengths of the clusters at the end and beginning of the experiment (Christensen *et al.*, 2015).

### Statistical analysis

The effects of *p*CO_2_ (three levels: 400-, 800- and 1200-μatm CO_2_) and seawater temperature (two levels: control-15°C and 24°C both for the highly acidified environment) on the mussel responses were tested at the cluster level. The effect of SW was only tested in the highly acidified scenario, assuming the most likely future ocean characteristics, i.e. more acidic and warmer simultaneously according to most predictive models. Such unbalanced experimental design does not allow us to establish potential interactions between *p*CO_2_ and temperature increase (no information was obtained for SW and actual *p*CO_2_ values). Therefore, two-way ANOVA analysis was performed with treatment (four levels: control, moderate acidification, strong acidification-cold water and strong acidification-warm water) and origin (Galician and Tunisian mussel populations) as the main factors. The CR data were subjected to repeated measures ANOVA. Differences between groups over time (2 months) were determined once a week (see Section 2.3.1). For the other mussel responses (valve opening behaviour, byssus strength, STI, shell compressive strength, OM of the shell and aragonite:calcite ratio, CI and SGR), the data were subjected to a two-way ANOVA to determine differences between treatments tested and mussel origin. Once the lack of any effect of the experimental unit (tank) was confirmed, the analyses were repeated for the factors under study. Normality and homogeneity of variance were examined by Shapiro–Wilk’s *W*-test and Levene’s test, respectively. Whenever the assumptions of analysis of variance were violated, data were log or rank transformed (Conover, 2012). Homogeneous groups were established *a posteriori* with the Bonferroni-adjusted level for distinct sample sizes and Tukey tests for multiple comparisons. All values shown in the figures are means ± SE. All analyses were performed with SPSS Statistics 23 (IBM) and STATISTICA v.7.0 software (StatSoft). Differences were considered significant at *P* < 0.05.

## Results

### Clearance rate

**Figure 1:**
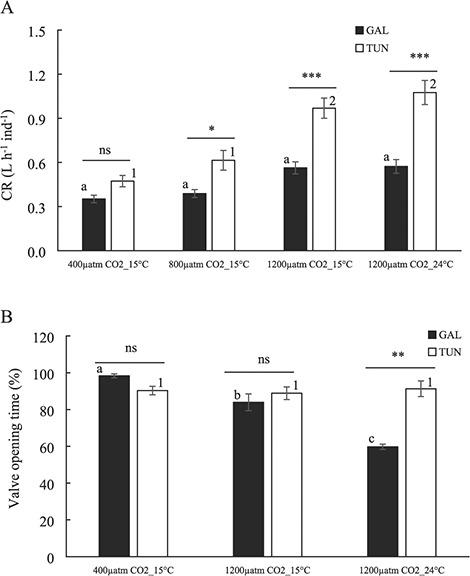
(**A**) CRs (L h^−1^ ind^−1^) and (**B**) valve opening time (%) in the mussels from both origins after exposure to increased *p*CO_2_ values and seawater temperatures. Both responses correspond to integrated values during the experimental period (2 months; see Materials and Methods). Black and white bars corresponded to Galician and Tunisian mussels, respectively. Values are means ±SE. Statistical analyses are included with letters (Galician population) and numbers (Tunisian population) to highlight differences between treatments (distinct letters or numbers mean significant differences). Horizontal lines above the plotted bars illustrate differences between populations (*P < 0.05; **P < 0.01; ***P < 0.001; ns, not significant).

The variation in the CR of the mussels in relation to the distinct scenarios of *p*CO_2_ and temperature conditions is shown in Fig. 1A. The results of the two-way ANOVA for CR variation are included in Table 2. Both treatment and mussel origin factors had a significant (*P* < 0.001) but interdependent impact on CR (*P* < 0.01; Fig. 1A; Table 2). The CR was significantly higher in mussels from the Mediterranean Sea (Tunisia) as compared with the Atlantic Sea (Galicia) especially for highly acidified environments regardless temperature value (0.97–1.08 L.h^−1^ vs. 0.56–0.57 L.h^−1^ for both Tunisian and Galician mussels, respectively; see interaction term treatment × origin; Fig. 1A). A slight increase of CR though not statistically significant was observed for Galician mussels with regard to acidification increase (0.35–0.56 L.h^−1^). By contrast, Tunisian mussels increased CR significantly with the highest acidified scenario from 0.47 L.h^−1^ under control scenario to 0.97 L.h^−1^. No effect of SW under highly acidified scenario was detected for any of the mussel populations on CR values (Fig. 1A).

**Table 2:** Results of repeated measurements ANOVA (CRs) and two-way ANOVAs to determine the effect of the factor treatment (four levels: 400-μatm *p*CO_2_/15°C, 800-μatm pCO2/15°C, 1200-μatm pCO2/15°C, 1200-μatm pCO2/24°C) and origin (two levels: Galician and Tunisian populations) on distinct mussel responses

CRs				Valves opening time_rank transformed			
Factor	df	F	*P*	factor	df	F	*P*
Treatment	3	35.660	**<0.001**	Treatment	2	7.083	**<0.01**
Origin	1	87.815	**<0.001**	Origin	1	1.587	ns
Treatment × origin	3	6.603	**<0.01**	Treatment × origin	2	10.848	**<0.001**
Error	16			Error	17		
**Byssus strength_rank transformed**				**STI_rank transformed**			
Treatment	3	1.181	ns	Treatment	3	0.247	ns
Origin	1	17.172	**<0.001**	Origin	1	364.139	**<0.001**
Treatment × origin	3	9.455	**<0.001**	Treatment × origin	3	1.816	ns
Error	142			Error	142		
**SCS_rank transformed**				**Shell OM_rank transformed**			
Treatment	3	15.629	**<0.001**	Treatment	3	0.787	ns
Origin	1	0.163	ns	Origin	1	15.216	**<0.001**
Treatment × origin	3	2.312	**~0.078**	Treatment × origin	3	0.601	ns
Error	141			Error	45		
**Ar:Ca ratio**				**SGR**			
Treatment	3	1.644	ns	Treatment	3	5.756	**<0.01**
Origin	1	5.880	**~0.027**	Origin	1	40.270	**<0.001**
Treatment × origin	3	1.279	ns	Treatment × origin	3	7.859	**<0.01**
Error	17			Error	16		
**CI_rank transformed**							
							
Treatment	3	18.743	**<0.001**				
Origin	1	234.953	**<0.001**				
Treatment × origin	3	5.376	**<0.01**				
Error	64						

When indicted, dependent variables were rank-transformed prior to the analysis. Tank was previously included in the analysis as random experimental unit (see Materials and Methods) but subsequently removed when confirming the non-significant effect.

Values in bold are statistically significant (*P* < 0.05); ns indicates not significant.

### Valve opening behaviour

The valve opening behaviour of the two origins of the mussels under the different *p*CO_2_ and temperature conditions tested is presented in Fig. 1B. The results of the two-way ANOVA for valve opening values as a function of treatment and mussel origin are included in Table 2. A treatment factor showed a significant effect on valve opening of mussels (*P* < 0.01) and the interaction term (*P* < 0.001) suggested that such effect depended on the mussel origin (Fig. 1B; Table 2). Indeed, highly acidified scenarios regardless temperature did not show any significant impact on valve opening time of Tunisian mussels (88.88–91.29%; Fig. 1B). By contrast, acidification (15%) and especially acidification plus warming (40%) both caused a significant decrease in valve opening time of Galician mussels (Fig. 1B).

### Byssus strength

**Figure 2:**
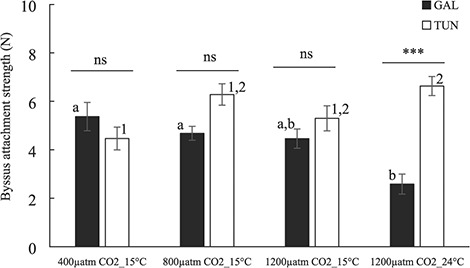
Byssus attachment strength (N) of the mussels from both origins after exposure to distinct *p*CO_2_ and temperature scenarios for 2 months. For correspondence between the colour of the bars and origin and statistical analyses, see the legend for Fig. 1. Values are means ±SE.

Byssus attachment strength of the experimental populations in the different tested scenarios is shown in Fig. 2 and two-way ANOVA results are presented in Table 2. The origin of mussels showed a significant effect on byssus strength (*P* < 0.001) but the interdependency between both origin and treatment factors was detected based on the interaction term effect (treatment × origin, *P* < 0.001; Table 2). Byssus strength showed a slight increase in Tunisian mussels with moderate and highly acidified scenarios (from 4.41 N to 5.76–6.22 N), whereas the opposite was noted for Galician mussels (from 5.30 N to 4.36–4.61 N) (Fig. 2) though statistical differences were not significant due to high variability in any case. Simultaneous highly acidified and warmer scenarios caused no effect for Tunisian mussels as compared with highly acidified and colder environment (5.76–6.60 N), whereas a 44% decrease in byssus strength was noted for Galician mussels despite high variability noted (Fig. 2).

### Shell characteristics: STI, strength, OM and aragonite:calcite ratio

**Figure 3:**
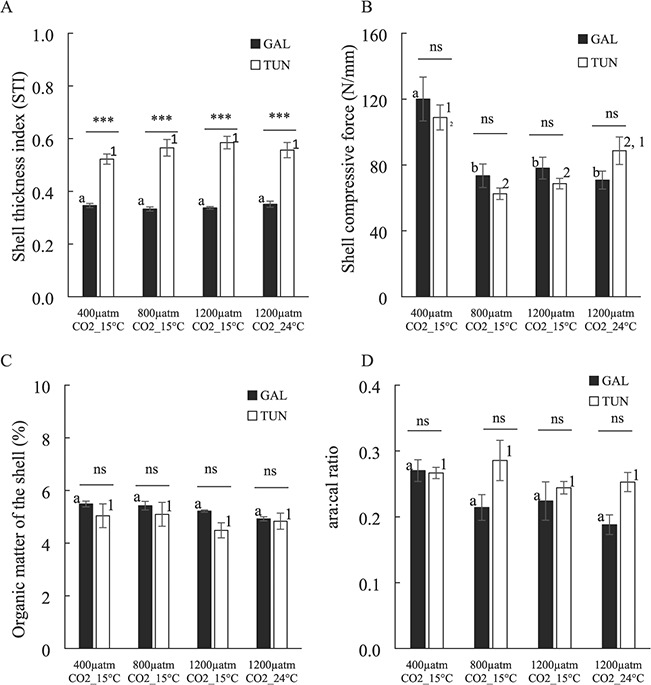
(**A**) STI, (**B**) shell compressive strength (N mm^−1^), (**C**) OM content of the shell (%) and (**D**) aragonite:calcite (Ar:Ca) ratio obtained for the shells of the experimental mussels after exposure to distinct *p*CO_2_ and temperature scenarios for 2 months. For correspondence between the colour of the bars and origin and statistical analyses, see the legend for Fig. 1. Values are means ±SE.

The variability in the STI as a function of treatment and mussel origin factors is shown in Fig. 3A. Two-way ANOVA showed that only mussel origin was significant explaining STI variability as independent factor (Table 2). Shells of Tunisian mussels were much thicker (0.52–0.59) than those of Galician mussels (0.33–0.35) when both factors were considered together (*P* < 0.001).

The compressive strength of mussel shells standardized by the variability in shell thickness varied significantly with the treatment considered but not with the mussel origin neither the interaction term treatment x origin though the latter factor represented a residual value (*P* = 0.078) (Table 2; Fig. 3B). Both mussel populations responded similarly to moderate and high acidified scenarios with a significant decrease in shell compressive strength (from 110–122 N.mm^−1^ in the control scenario to 63.2–78.7 N.mm^−1^; Fig. 3B) considering both mussel populations.

Galician mussels maintained under a highly acidified and warmer environment showed no significant change in shell compressive strength with regard to a highly acidified and colder environment (Fig. 3B). By contrast, Tunisian mussels showed a slight increase (28%) in shell compressive strength for the same treatment comparison, which caused that minor statistical differences were noted with regard to highly acidified and colder but also the control environments, respectively (Fig. 3B).

Variability in the OM of the shell for the two mussel populations exposed to distinct *p*CO_2_ and temperature environments is presented in Fig. 3C. Only the mussel origin was shown to present a significant impact on OM of the shell but not the treatment neither the interaction term (see two-way ANOVA in Table 2) despite the fact that shell OM decreased slightly in the Tunisian mussels under the low temperature, high acidification conditions (11%) and in the Galician mussels under high temperature, high acidification conditions (6%) (but note the high variability in all cases; Fig. 3C). The OM content of the shell (periostracum) showed mean values of 5.3 and 4.8% for both Galician and Tunisian populations including all treatments tested.

The variability in the ara:cal ratio in response to the different scenarios is shown in Fig. 3D. No effect was observed for the treatment factor despite a slight decrease obtained in ara:cal ratio values especially for Galician mussels subjected to moderate and highly acidified (both cold and warm) scenarios (20–30% but note the high variability in Fig. 3D). Mussel origin was the only factor showing a residual effect (*P* ≈ 0.027), the Tunisian mussels having slightly greater ara:cal ratio values (0.26) than Galician mussels (0.22) as means for all treatments (Fig. 3D; Table 2).

### CI and SGR

**Figure 4:**
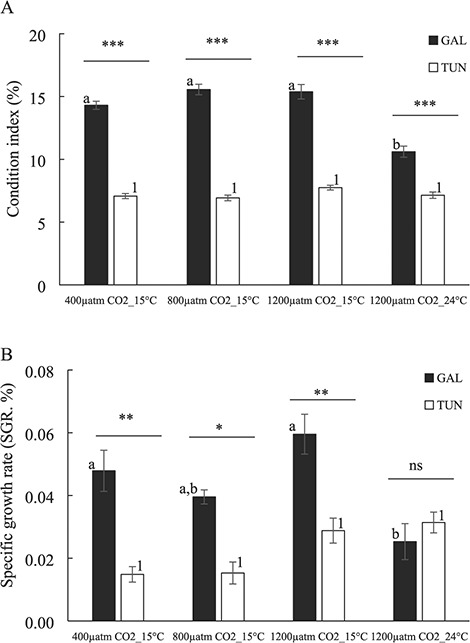
(**A**) CI (%) and (**B**) SGR (%) in the mussels from both origins after exposure to high seawater temperature and *p*CO_2_ for 2 months. For correspondence between the colour of the bars and origin and statistical analyses, see the legend for Fig. 1. Values are means ±SE.

CI variability with regard to *p*CO_2_ and temperature treatments considering the two mussel populations and the corresponding two-way ANOVA analysis are presented in Fig. 4A and Table 2, respectively. Both treatment and origin (*P* < 0.001) showed a significant effect on CI variability but such impact was interdependent (see the interaction term treatment x origin; *P* < 0.01) (Fig. 4A; Table 2). Galician mussels showed greater CI values as compared with Tunisian mussels and similarly for control, moderate and highly acidified-cold scenarios (14.30–15.56% and 6.92–7.74% for Galician and Tunisian populations, respectively; Fig. 4A). The exposure of Tunisian mussels to highly acidified and warmer environments did not change CI values but such scenario caused a significant decrease in CI for Galician mussels with regard to highly acidification alone (from 15.38% to 10.60%; Fig. 4A).

As was the case of CI, SGR showed also differences by treatment (*P* < 0.01) and origin (*P* < 0.001) factors that were in turn interdependent (*P* < 0.01; see Fig. 4B and Table 2). Galician population grew at greater rates as compared with Tunisian mussels with no significant differences among control, moderate and highly acidified-cold scenarios (0.040–0.060 and 0.015–0.029 for Galician and Tunisian populations, respectively; Fig. 4B). Highly acidified and warmer scenario caused no significant effect for Tunisian mussels despite slight increase in SGR at both highly acidified environments (cold and warm) with regard to control environment (Fig. 4B). Galician population subjected to such highly acidified and warmer environment showed a significant decrease in SGR with regard to highly acidified-cold test (from 0.060 to 0.025; Fig. 4B).

## Discussion

In the present study, the simulated scenarios of OA and SW for the highly acidified condition had very different effects as climate change drivers, with some independent and additive effects on mussels’ responses during the 2-month-long study. Regarding the behavioural and eco-physiological parameters considered, an interesting range of responses was observed in the mussel *M. galloprovincialis*, originally from very different, widely separated and heterogeneous environments (Tunisia, in the Mediterranean Sea and NW Spain in the Atlantic Ocean). Overall, highly acidification and SW together not only affected negatively the Galician population, namely in terms of abrupt reduction in the valve opening, CI and shell growth, but also weakened the attachment strength, though high variability for this response gave minor statistical differences between highly acidified cold and warmer scenarios. By contrast, OA alone had very different effects on mussel responses, ranging from positive (increased CR as indicative of higher feeding activity especially for Tunisian population) to negative (mainly shell strength for both mussel populations and, in a lower magnitude, valve opening time for the Galician mussels). Acidification alone did not have any statistically significant effects on shell characteristics such as thickness, OM or ara:cal ratio but neither on the state of soft tissues (CI), byssus strength and SGR as common response to both Tunisian and Galician populations most likely due to high variability noted within treatments. Moreover, acidification alone showed no clear effect on CR of Galician mussels and valve opening of Tunisian mussels, highlighting the interdependency of both factors (treatment and origin) for these mussel responses. No mortality was observed during the experiment, and the reported changes can therefore be considered to correspond to sub-lethal effects of the climate change drivers investigated.

OA is known to imply changes in energy allocation patterns to essential physiological processes of marine calcifiers, such as acid–base homeostasis, negatively affecting growth, reproduction and defence mechanisms (Kroeker *et al.*, 2014). In the previous studies of the Mediterranean mussel *M. galloprovincialis*, the negative effect of OA on growth performance occurred under much lower pH values than proposed here (see Range *et al.*, 2014). By contrast, other studies have highlighted either neutral, which established a rather resilient behaviour, or even positive effects of OA on metabolism and growth of calcifiers (Fabry *et al.*, 2008; Ries *et al.*, 2009; Fernández-Reiriz *et al.*, 2012; Gazeau *et al.*, 2014; Mackenzie *et al.*, 2014; Range *et al.*, 2014). Such resilience to OA may be due to local adaptation of the individuals to habitats such as estuarine and upwelling zones, in which natural food resources are abundant (see Duarte *et al.*, 2014; Lagos*et al.*, 2016), and cold, CO_2_-rich waters (Feely *et al.*, 2008). This would explain the resilient character of the Galician population (NW Spain), which is subjected to the influence of the Eastern North Atlantic Upwelling System. Nevertheless, the Tunisian population, far from this upwelling system of the Atlantic and originally from more vulnerable areas in terms of OA (Flecha *et al.*, 2015), was also not affected by moderate or strong acidification and was able to grow (see mean values for highly acidified scenario despite high variability), as was the Galician population, although to a lesser extent (and under-saturated values relative to aragonite ΩAr < 1.0).

The mineral phase of the shells of calcifiers is expected to be eroded under OA, and energy uptake may therefore be crucial to maintain integrity and functionality (Fitzer*et al.*, 2015). In the present study, the CR of mussels in both populations increased in response to acidification though only statistically significant for Tunisian population, probably due to the optimal diet supplied and the distinct necessity to respond to OA stress. However, shell strength of juveniles was consistently and similarly reduced in response to both intermediate and strong acidification of seawater. Weakened shell was also noted in other studies either as more brittle outer shells or softer aragonite inner shells (Gaylord *et al.*, 2011; Li *et al.*, 2015). Juvenile individuals of the same species (*M. galloprovincialis*) showed similar weakening of shell strength, regardless of whether the diet was optimal or sub-optimal (Lassoued *et al.*, 2019). At the end of the present study, mussels had grown (positive SGR values for both populations), indicating that their ability for calcification remained active, even in under-saturated conditions (ΩAr < 1.0) when optimal diet was supplied (see also Thomsen and Melzner, 2010; Thomsen *et al.*, 2010), although this may have occurred at the cost of shell structural integrity (see also Melzner *et al.*, 2011).

In the search for potential causes of shell weakening in response to seawater acidification (~40% for both populations), none of the characteristics monitored in the present study (shell thickness, OM or ara:cal ratio) provided insight into the weakening response. The high variability in the responses to each experimental treatment resulted in no statistical differences, despite a slight decrease in OM, i.e. periostracum (5–11%; see also Gazeau *et al.*, 2014: Li *et al.*, 2015) and ara:cal ratio (17–20%). Similarly, Mackenzie *et al.* (2014) showed that the variability in thickness and ara:cal ratio (unchanged) after 6 months under acidified and warmer scenarios in the mussel *M. edulis* did not explain the decrease in shell strength, which was more affected by warming than by acidification. The importance of OM in protecting the shell structure against OA has been demonstrated (Ries*et al.*, 2009) and although relatively small changes in this layer can strongly impact shell performance, a more precise micro-analysis is necessary to identify such patterns. Changes in the orientation of the mineral units that form the shell, modification of size and elongation of prismatic structure as well as decreased surface area to volume ratio may help to explain the increased fragility through a greater shell corrosion or a reduced microstructural complexity (Mackenzie *et al.*, 2014; Milano *et al.*, 2017). Although these aspects require further investigation, whatever the cause of the shell weakening, the fragility of the shells of juvenile *M. galloprovincialis* represents a significant threat for their survival potential and for ecological interactions within communities (e.g. predatory actions, competition).

Examination of the effects of combinations of eco-physiological and behavioural responses such as byssus attachment strength, feeding activity and valve opening allowed us to observe the potential impact of OA on primary activities that are important for the optimal development and survival of a sessile and gregarious species such as *M. galloprovincialis*, with implications on the ecology and cultivation of the species. OA negatively affects the attachment strength of marine mussels (O’Donnell *et al.*, 2013; Zhao *et al.*, 2017); although, Clements *et al.* (2018) noted that this pattern is not universal, e.g. it will depend on other factors such as CI. Indeed, we have recently reported the absence of any effect of OA on byssus attachment strength of *M. galloprovincialis* juveniles, when fed optimally (Lassoued *et al.*, 2019). In other words, optimally fed individuals would be able to cover the energy-demanding biological mechanisms to cope with OA stress. The attachment strength of the mussels did not change under the acidified conditions and regardless of origin in comparison to control scenarios. Thus, both the Atlantic and Mediterranean populations of this species appear to be rather resilient in relation to such key environmental driver, with important implications for the impact of future competitive performance and cultivation of the species. Byssus secretion depends on the ability of the mussels to open the valves to extend the foot for chemotaxis. A neutral effect of acidification on attachment strength was observed, despite valve opening decreased slightly, e.g. 15% in the Galician mussels. By contrast, the slight increase in CR or feeding activity for the Galician population (though not statistically significant) under moderate and high acidified conditions may have complemented with the energy needed for maintaining optimal byssogenesis rates and consequently strength and even allowed a positive shell length growth (see above).

Upper temperature threshold for optimal metabolism and performance of *M. galloprovincialis* mussels was set at 24–25°C (Anestis *et al.*, 2007, 2010), since mortality increased significantly above 25°C, with peaks at 28°C (summer) after 10 months exposure (Gazeau *et al.*, 2014). Overall, elevated temperature may produce a cascade of metabolic events such as alteration of the cell membrane permeability and disruption of acid–base homeostasis caused by OA, thus affecting defensive patterns or leading to metabolic disturbance (Pörtner, 2010) and also impacting on energetic trade-off patterns. In the present survey, we focused on potential additive effects between warming and strong OA (for reviews, see Orr *et al.*, 2005, Feely *et al.*, 2008, and Gazeau *et al.*, 2013), which will depend on the thermal window of marine organisms (Pörtner and Farrell, 2008), very distinct for the Atlantic and Mediterranean populations under study (see Materials and Methods).

The Tunisian population of mussels was not negatively affected by the higher seawater temperature tested in addition to highly acidified scenarios (see all behavioural and eco-physiological indices reported here). In view of the temperatures that this population can resist in summer (see Materials and Methods), 2 months of exposure to high-temperature experimental conditions (24°C) did not imply a serious threat. According to our results, the negative effect of a highly acidified environment on shell strength could be partly ameliorated by SW only for Tunisian mussels though such increase in shell strength (22%) with regard to highly acidified and cold scenario was sufficient to represent no statistical differences with the control scenario. Changes in the water chemistry in relation to saturation states of the carbonates between colder and warmer highly acidified scenarios would help to explain such partly counterbalance for shell strength (Table 1; see also Gazeau *et al.*, 2014; Kroeker *et al.*, 2014). Similarly, the impact of OA on other shell characteristics of marine calcifiers (such as shell dissolution, size and calcification rates) may be ameliorated at higher temperatures (Thomsen *et al.*, 2013; Lagos *et al.*, 2016). By contrast, the negative impact of OA alone on Galician population was significantly amplified by the elevated temperature, as reflected in the abrupt decrease in valve opening time (much greater than under OA alone), byssus strength (halved that under acidic conditions but not statistically significant due to high variability), CI (due to intermittent spawning events observed in the tanks during the experiment) and eventually growth (SGR) and, to a much lesser extent (not significant), shell strength or ara:cal ratio. The highly acidified environment caused a reduction of 15% of the valve opening time of Galician mussels (see above) but this reduction reached 40% at the higher temperature (and highly acidified) conditions (additive effect). It can be hypothesized that mussels exceeded the threshold of opening time to behave optimally regarding foot extension and therefore, byssus secretion (but not feeding activity). Shell characteristics monitored here for the Galician population were not significantly affected by the simultaneous higher temperature and highly acidified scenario (see shell strength and thickness, OM or ara:cal ratio) though there was a tendency for the mean values to slightly decrease in all cases for that most stressful treatment. This aspect would need more investigation with elevated temperature tests alone to monitor the actual synergies (not explored here) between both OA and warming, e.g. elevated temperature may have had an effect on shell microstructure, changing crystals width, thickness or angle of spread in the nacreous structure that needs to be confirmed (see Olson *et al.*, 2012).

In summary, our findings showed that the original habitat greatly affected the ability of the mussel *M. galloprovincialis* to cope with key stressors involved in climate change. Although it is difficult to determine the impact of future environmental scenarios, because adaptive responses (genotype and phenotype) will occur, the present findings highlighted that OA process alone and potential additive effects between acidification and SW are likely to have ecological and functional implications for the populations. Testing the effects of elevated temperature in a strongly acidified environment is important considering the present high values recorded in seawater in NW Spain (Galicia) in the summer periods (up to 20°C) and the worst scenario projected by the IPCC. Both stressors together had drastic eco-physiological effects on the Atlantic population, i.e. shortened valve opening time, weakened strength of attachment and decreased CI and shell growth. The acidified conditions alone caused a consistent decrease in shell strength regardless of mussel origin. The impact on shell strength represents, therefore, a risk to both the Atlantic and Mediterranean populations, with regard to the ecological implications of a weaker protective shell. Any reduction in mechanical strength or changes in shell microstructure and composition may have profound impacts on survival, either by reducing protection of vital soft tissues or shifting the ability to respond to environmental change (Mackenzie *et al.*, 2014). Mussels probably require compensatory adjustments (energetically costly) to repair shell functionality, and the Atlantic population may not be able to afford these adjustments because the simultaneous temperature increase may reduce the endogenous reserves (see CI decrease), which may also have other effects on, e.g. fecundity.

Future predatory actions in the community or high-energy environments may affect the adaptive potential of this species under the projected OA. Changes in the ability of the individuals to produce a strong shell, as key protective tissue, by OA would be the most critical aspect for future aquaculture perspectives and the community level effects. In case of farmed mussels, operational mechanical actions such as separation of clusters by removal of the byssus and cleaning attached fouling on the shell may lead to loss of marketable product due to shell breakage that can represent up to 5–15% of the whole annual production (G. Sarà, personal communication in Martinez *et al.*, 2018). Cracked mussels are unwanted products for the market and represent a production loss with the consequent impact in the aquaculture sector. At community level, effects may be reflected in the reduction of shell and skeleton formation, shifting or loss in biodiversity, and due to the key role of mussels, as ecosystem engineers and habitat-forming species (through their cluster-forming capacities), significant changes in these types of natural assemblages and food webs. These negative perspectives linked to shell production under OA scenario were clearly shared by the two mussel populations (Atlantic and Mediterranean) under the study. Besides, a serious threat for the future yield values of this cultivated species, especially at the Atlantic side and the cultivation system itself, is related to the potential unstable attachment secreted by the individuals on cultivation ropes when acidification and warming are combined together plus a reduction in soft tissues and shell growth. Mussel seed mortalities that are emerging as relevant threat for the aquaculture sector in recent years may be related to the shift in such attachment capacities of individuals. The importance of associated fauna that coexist with the cultivated biomass and occasionally may bring negative consequences on the maintenance of individuals attached on aquaculture systems (Babarro*et al.*, 2019) may play a key role on the eventual decrease in biomass produced. Shifting in the ecological balance of these aquaculture systems with the accompanying fauna may alter labour and yield aspects of the aquaculture sector as well as threaten especially the development of marine monocultures in the near future. In addition, biodiversity may be altered as a consequence of shifts in the abundance of the mussels that can locally differ due to specific environmental characteristics and changes as illustrated here. New perspectives for future aquaculture activities should extend this type of research by using simple approaches to highlight the consequences of the predicted environmental change and the actual potential of marine calcifiers to cope with change. The responses of target organisms to natural and predicted environmental heterogeneity represent the basis for understanding and protecting natural resources and may have a significant impact on socio-economical services.
